# Indents

**Published:** 1906-11-15

**Authors:** 


### INDENTS.

☞ **Brief Articles of Technical or Scientific Value will receive notice and comment. Contributions invited.**

**High Pressure Desensitizing.** Use a No. 1-2 round bur drilling with a light touch until the slighest sensation is felt, then take a No. 2 bur and give just the slighest countersink to the cavity, and then apply your high pressure.

In drilling the initial pit, stop and apply high pressure the moment the slighest sensation is experienced. After a few seconds remove the syringe and deepen the pit until sensation is felt, when a re-application of pressure anaethesia is made for a short time, and again drill till sensation is produced or until the pulp chamber is approached very closely if it is desired to remove the pulp, in this case high pressure should be continued for some time prior to entering the chamber if the pulp is to be removed without causing pain.

If on the other hand, it is a sensitive cavity that is to be prepared then drill only deep enough into the tooth to get a good adjustment of the point, and at the same time reduce the resistance in the tooth substance till the solution may be forced deep enough to permit the preparing of the cavity without causing pain.

No rule can be laid down giving either the depth of the pit or the length of time high pressure must be continued to procure results. The age of the patient, the size, condition and position of the cavity aids the experienced operator in judging this to a certain extent, but it is only the actual test which enables one to tell when sufficient aneastheasia has been produced. The test the writer uses is to drill into the cavity a pit, or if a larger cavity to cut out and prepare that part of the cavity which is most removed from the point at which the injection is made. When that can be done, then it is afe to destroy your initial pit and proceed with the cavity preparation

There is no more excuse for injuring or endangering the life of the pulp in the use of this method than there is for the physician to injure or endanger the life of his patient in the administration of any of the many poisons that are used thousands of times daily in the treatment of the various diseases. W. G. Ebersole in *Dentists Magazine*.

**Plate Retention.** Every dentist knows that the center of the palate in 98 per cent of jaws is hard, but few seem to realize the fact that this portoin of the jaw never changes, while the alveolar ridge is subject to constant change to greater or less extent, especially is this true in the wearing of a vulcanite plate, which in consequence of its non-conductibility causes in 80 per centof jaws excessive resorption and this is a serious objection to the use of vulcanite on the upper jaw for permanent dentures. It should be the duty of every dentist to inform his patients of this fact. The change under metal, as a rule is very little.

Unless provision is made for this change in the alveolus, sooner or later the plate is rocking over the hard center. The provision which I make for this is to place what I call a "relief" over the hard surface. This is done with a thin base plate wax covering it about one-half inch according to width of jaw and extending to near top of ridge and to within $\frac{3}{16}$ inch of posterior margin of the plate. Plates can be worn farther back than usually made. The margins of the relief to be thinned flushed with the plate, no defined edge.

In case of vulcanite either scrape the impression or use sheet lead, same thickness of the wax and cement it to the model. The serious objection to the patent suctions so extensively used is that they necessitate using the vulcanite plates for permanent work and mischief enough has already been done with this material as appears from the thousands of flat, ridgeless jaws.

As an evidence that the vacuum cavity is not at all needed I cite a set of teeth on aluminum made for a patient eleven years ago.

It was such a peculiar case that I made a set to keep as a curiosity. It and the cast has been seen by hundreds of dentists who wondered how it could be retained on the jaw. What ridge did exist was flexible. On the right side a portion of the bone had been removed so that is hard, and the palatal bone is hard and elevated. The small section of the ridge which was hard was relieved so the plate would not bear hard as the soft margin yielded to pressure, the center has the usual relief. The unusual length of bite was needed in order to restore the proper expression.

The patient called on me one year ago and said he often forgot he was wearing teeth, and that his "set was a daisy"

I called a dentist to examine it in his mouth and he found it required an effort to remove it.

Better results are secured in these flat ridgless jaws, with the swaged metal plate than with vulcanite. If the vacuum cavity is not needed in these cases, where is it needed?—L. P. Haskell in *Dentists Magazine*.

**Retention of Artificial Dentures.** What are the objections to these patent suction forms? (*a*) They are made of a material that is soon destroyed by the fluids of the mouth. (*b*) The material and its form is very unhygienic, the most so of anything we use in the mouth.

Within a few months, flexible vulcanite will become saturated with the oral fluids, and if the patient neglects to change it often, it will not only add its peculiar offensive taint to the breath, but the incerased bulk of the diaphram will interfere with the retention of the denture. (*c*) Dr. Haskell says:—"The serious objection to the patent suction so extensively used is that they necessitate using the vulcanite plates for permanent work; and mischief enough has already been done with this material as appears from the thousands of flat ridgless jaws". (*d*) The necessarily thickened plate. (*e*) Last but not least, is the effect the general use of these suction chambers have upon the prosthetist. Their general use will tend to make us a stereotype mechanic. All cases will look alike to such an one. We should study and diagnose each case; if we do not, we are in truth dental mechanics.

Second: What are the conditions of the mouth that are favorable and unfavorable for retention of artificial dentures.

(*a*) Size: All things being equal the larger the jaw the greater the retention. (*b*), Shape of the vault: A very high sharp peaked vault or a very flat one are unfavorable, while a medium high oval vault is the most favorable. Of the four conditions of the mouth we are now considering, the shape is of the least consequence. (*c*) Soft tissues: The soft tissues require careful consideration, for they are very important. We may have a thin tense mucous membrane over the whole jaw: This would be the logical place for use of a flexible suction chamber. We may have an excessive layer of soft tissues over the whole jaw. In this case a flexible suction chamber would be a positive detriment. And then we may have a variety of combinations of the tense and flabby soft tissue. In these cases the skilful experienced man will render better service without flexible suction chambers; although they may be used with *apparent* success. (*d*) Fluids of the mouth: The fluids of the mouth are of equal importance with the condition of the soft tissues. If the fluids are normal as found in a healthy young person they are most favorable for adhesion by contract, but if the fluids are thick, viscid and ropy they are most unfavorable.

Thus a most unfavorable case would be, a small jaw with either a high peaked or flat vault, covered with a thick layer of fissured soft tissue and bathed in an excessive amount of thick viscid ropy secretions. If the patent suction chamber would help in these most unfavorable cases they would indeed be a blessing; but they will not.

There are other factors that must be considered in diagnosis and prognosis. We will name them but not discuss them. Relation of the jaws to each other (leverage). State of the general health. Temporary exhaustion. The ability of the patient to adapt himself to environments. The mind of the patient must always be taken into consideration.

Third: What is the philosophy of retention by a flexible suction chamber?

Full upper dentures are retained by two forces. *First*, atmospheric pressure, which is caused by a cavity in which there is a partial exhaustion of the atmcsphere. This force exists only so long as there is a space in which there is more or less rarified air. As soon as the space is filled with soft tissue and the fluids of the mouth, atmosphereic pressure ceases; and the second force, adhension by contract, is the retaining force. These forces must not be confused with each other, because they are entirely different. Atmospheric pressure always implies a cavity from which the air is partially exhausted. Adhesion by contact means absolute contact. Thus if we take two flat smooth pieces of glass and place them together we would not have adhesion because there would be a slight film of air between them, but, if we moisten the surfaces and press them closely together we will have considerable adhesion. This is not atmospheric pressure because by the hydrostatic law the air pressing upon the periphery of film of water distributes the pressure in every direction; thus the glass platss will be held together by the strength of the film of water. As attraction and repulsion are nearly equal in water the thinner the film the greater the attraction; consequently the thinner the film of water, the stronger will be the attachment of the glass plates.

Let us apply these principles of physics to the soft rubber disk. We assume the rubber has been well vulcanized and is not of itself sticky or adhesive. We can readily see how it is much easier to get absolute contact with velum vulcanite to an uneven hard surface than it is with hard vulcanite.

We also see that the adhesion by contact of the soft vulcanite is not very strong, because one portion of soft vulcanite gives but little support to another while a rigid material does.

When traction is applied upon the center of the disk it yields without displacing the periphery of the disk; but we have produced a new condition under the center of the disk a vacuum thus we have atmospheric pressure, suction real suction with its attendant evils upon the soft tissues underneath the disk. We have more than this, we have displaced the plate slightly from the rest of the jaw, and it simply hangs suspended. When visiting the supply house again, take their sample base plate and suction form and study its action on the glass model.

Fourth: Why do not dentures adhere better? (*a*) Because the case is not properly diagnosed. (*b*) Because of imperfect manipulation on the part of the dentist. (*c*) Because the patient has not the ability to adapt himself to the best conditions the dentist can produce. (*d*) There are a few cases (probably) that our known methods are not equal to the demands. (*e*) There are some cases, which because of the incompatible natures of prosthetist and patient, make it impossible for the operator to produce his best work and for the patient to co-operate. Such patients would probably meet with satisfaction at the hands of another though a less expert prosthetist.

We must say a few words upon division (*b*)of this fourth class. There are few men who take a really good impression.

This subject will well repay careful study. Most of the misfits that occur in vulcanite work, after a perfect impression is obtained are due to faulty packing and closing the flask. It is a mistake to attribute our failures to the materials used, it is rather our improper use of them.—G. H. Wilson in *Dentists Magazine*.

